# A Case Series of Thromboelastography-Guided Anticoagulation in COVID-19 Patients with Inherited and Acquired Hypercoagulable States

**DOI:** 10.1155/2021/5568982

**Published:** 2021-07-31

**Authors:** Anthony V. Thomas, Kevin P. Lin, John E. Stillson, Connor M. Bunch, Jacob Speybroeck, Grant Wiarda, Hamid Al-Fadhl, Laura Gillespie, Mahmud Zamlut, Daniel H. Fulkerson, Rashid Z. Khan, Hau C. Kwaan, Mark M. Walsh

**Affiliations:** ^1^Indiana University School of Medicine South Bend Campus, South Bend, IN, USA; ^2^Departments of Emergency and Internal Medicine, Saint Joseph Regional Medical Center, Mishawaka, IN, USA; ^3^Department of Quality and Performance Improvement, Saint Joseph Regional Medical Center, Mishawaka, IN, USA; ^4^Department of Intensive Care Medicine, Saint Joseph Regional Medical Center, Mishawaka, IN, USA; ^5^Department of Neurosurgery, Saint Joseph Regional Medical Center, Mishawaka, IN, USA; ^6^Department of Hematology, Michiana Hematology Oncology, Mishawaka, IN, USA; ^7^Division of Hematology and Oncology, Northwestern University Feinberg School of Medicine, Chicago, IL, USA

## Abstract

One of the complications of the novel coronavirus disease 2019 (COVID-19) is hypercoagulability. For this reason, patients presenting with COVID-19 are often put on therapeutic or intermediate anticoagulation upon hospitalization. A common issue of this anticoagulation is the progression to hypocoagulability resulting in hemorrhage. Therefore, monitoring the hemostatic integrity of critically ill COVID-19 patients is of utmost importance. In this case series, we present the cases of three coagulopathic COVID-19 patients whose anticoagulation was guided by thromboelastography (TEG). In each case, TEG permitted the clinical team to simultaneously prevent thrombotic and hemorrhagic events, a difficult task for COVID-19 patients admitted to the intensive care unit. The first two cases illustrate the utility of TEG to guide anticoagulant dosing for COVID-19 patients when the activated partial thromboplastin time (aPTT) is inaccurate. The first case was a severely ill COVID-19 patient with end-stage renal disease and a falsely elevated aPTT secondary to hypertriglyceridemia. The second case was a severely ill COVID-19 patient with chronic pulmonary disease who demonstrated a falsely elevated aPTT due to polycythemia and hemoconcentration. In both cases, TEG was sensitive to the hypercoagulability caused by the metabolic derangements which enabled the goal-directed titration of anticoagulants. The last case depicts a severely ill COVID-19 patient with an inherited factor V Leiden mutation who required abnormally high dosing to achieve therapeutic anticoagulation, guided by TEG. Hypercoagulopathic COVID-19 patients are difficult to anticoagulate without development of hypocoagulopathy. Treatment of these patients demands goal-directed therapy by diligent laboratory monitoring. This can be accomplished by the use of TEG coupled with aPTT to guide anticoagulation. This case series illustrates the necessity for active hemostatic monitoring of critically ill COVID-19 patients.

## 1. Introduction

There has been dispute regarding the anticoagulation of patients diagnosed with the novel coronavirus disease 2019 (COVID-19) [1]. Initially, there was support for anticoagulation due to the high rates of thrombosis in COVID-19 patients in the intensive care unit, but standard doses of low-molecular-weight heparin and unfractionated heparin (UFH) may lead to hemorrhage in these patients [2–7]. A recent large United States trial regarding therapeutic anticoagulation of critically ill COVID-19 patients was discontinued because of the high rate of hemorrhage [8]. Determining a standard for anticoagulation was an attractive goal for the study, but the high doses of anticoagulation had proven to be potentially lethal.

These high rates of clotting and hemorrhage in COVID-19 patients prompted us to assemble a coagulation committee. This committee's goal was to prevent thrombosis in COVID-19 patients while simultaneously reducing the occurrence of clinically significant hemorrhage. Thromboelastography (TEG) was an appealing test for hemostatic monitoring due to its proven benefits in extracorporeal membrane oxygenation (ECMO) and trauma and its recent use in other studies regarding anticoagulation of hypercoagulopathic COVID-19 patients [9–13]. This case series illustrates the use of TEG in administering anticoagulation of three COVID-19 patients and preventing hemorrhage (Table 1). No written consent has been obtained from the patients as there is no patient identifiable data included in this case series.

## 2. Case 1: TEG-Guided Anticoagulation in a COVID-19 Patient with Falsely Elevated aPTT Caused by Hypertriglyceridemia

**Figure 1:**
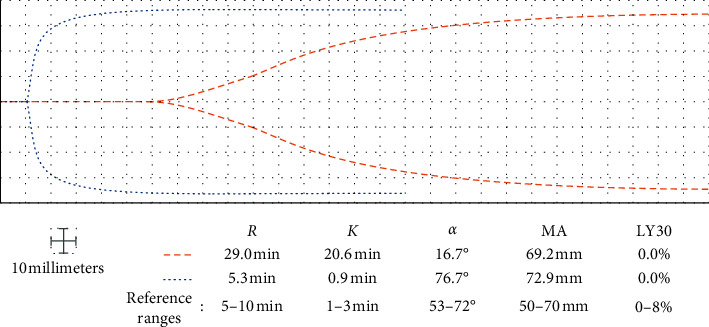
Nine days after admission, the aPTT and TEG concurred with a supratherapeutic hypocoagulable state, and the heparin drip was stopped (dashed lines). The next day, TEG demonstrated a hypercoagulable state and aPTT was discovered to be falsely prolonged due to the hypertriglyceridemia (dotted lines). *R*: reaction time; *K*: clot formation time; *α*: alpha angle; MA: maximum amplitude; LY30: percent lysis at 30 minutes.

A 24-year-old Hispanic male with a 2-year history of end-stage kidney disease requiring dialysis was hospitalized with critically severe COVID-19 pneumonitis. At admission, Doppler ultrasound of all four extremities was negative for deep vein thrombosis (DVT). He was administered 5,000 units of subcutaneous (SQ) UFH every eight hours for DVT prophylaxis. In addition, he received an intravenous (IV) UFH infusion because of the recent history of clotting dialysis lines. Seven days after admission, because of respiratory insufficiency and deterioration, he was endotracheally intubated and mechanically ventilated. Nine days after admission, he had an activated partial thromboplastin time (aPTT) of 50.2 seconds; however, TEG demonstrated excessive hypocoagulability with a prolonged reaction time (*R*) (Figure 1). His heparin drip was stopped, and the overall dose was decreased to include only 5,000 units SQ UFH every twelve hours. The following morning, TEG was consistent with hypercoagulability, but the aPTT measured 122.6 seconds by optical clot detection and 81.2 seconds by mechanical clot detection (measured using the Sysmex CA-1500 with reagents Innovin and CaCl_2_, Siemens Medical Solutions, Malvern, Pennsylvania, USA) (Figure 1). Triglycerides were 1,456 mg/dL and D-dimer was trending upward. It was discovered that the aPTT measurement by optical clot detection is only accurate to triglyceride levels <331 mg/dL according to the manufacturer, and thus, aPTT was falsely prolonged due to the lipemia. For the rest of his stay, TEG guided anticoagulation dosing of SQ UFH ranging between 5,000 and 7,500 units every eight hours. TEG enabled an individualized regimen to prevent clotting and hemorrhage from dialysis lines despite the hemostatic dysfunction from COVID-19 superimposed with hypertriglyceridemia and renal failure.

## 3. Case 2: TEG-Guided Anticoagulation in a COVID-19 Patient with Falsely Elevated aPTT Caused by Excessive Hemoconcentration

**Figure 2:**
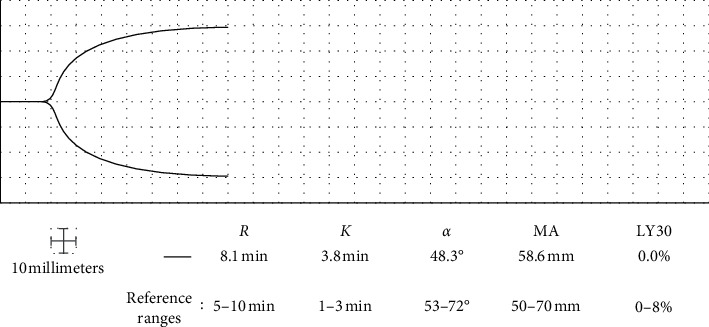
Nine days after admission, TEG demonstrated subtherapeutic anticoagulation in the setting of prolonged aPTT because of hemoconcentration and polycythemia. *R*: reaction time; *K*: clot formation time; *α*: alpha angle; MA: maximum amplitude; LY30: percent lysis at 30 minutes.

A 66-year-old Caucasian male with a history of chronic obstructive pulmonary disease and DVTs on chronic rivaroxaban was admitted with a moderately severe COVID-19 pneumonitis and appropriate secondary polycythemia. Doppler ultrasound of the extremities was negative for DVT at admission. Home rivaroxaban was stopped, and he was started on 9 units/kg/hr IV UFH for DVT prophylaxis. Two days after admission, he was started on furosemide 40 mg IV every twelve hours for pulmonary edema. Nine days after admission, the polycythemia was worsened to a hemoglobin of 21.4 g/dL, likely due to hemoconcentration and the COVID-19 inflammatory response. Titration of UFH was guided by aPTT every six hours and daily TEG. A sudden increase in the aPTT to 82.3 seconds occurred when the patient's polycythemia increased beyond 21.4 g/dL. Based on the elevated aPTT, the clinical team reviewed the patient's TEG tracing which demonstrated a lack of full anticoagulation despite an elevated aPTT (Figure 2). Review of the patient's rising hemoglobin and of the effect of the hemoconcentration on the aPTT revealed that, for hemoglobin more than 21 g/dL, the presence of citrate in the sample causes false elevation, rendering the aPTT ineffective in guiding anticoagulation in this patient. Thereafter, TEG guided UFH dosing between 7 and 9 units/kg/hr to achieve a target *R* of 12–17 minutes because the secondary polycythemia produced an inaccurate aPTT.

## 4. Case 3: TEG-Guided Anticoagulation in a COVID-19 Patient with Inherited Hypercoagulable State

**Figure 3:**
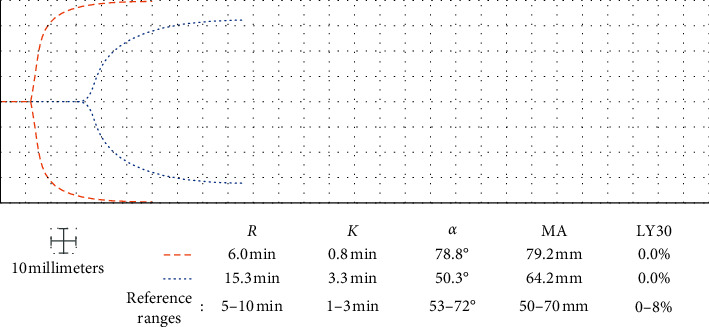
One day after admission, despite prophylactic anticoagulation dosing, a patient with factor V Leiden mutation demonstrated borderline hypercoagulability on his TEG tracing (dashed lines). Persistent heparin resistance prompted a dose increase to achieve therapeutic anticoagulation on TEG (dotted lines). *R*: reaction time; *K*: clot formation time; *α*: alpha angle; MA: maximum amplitude; LY30: percent lysis at 30 minutes.

A 49-year-old Caucasian male with a factor V Leiden mutation was admitted with COVID-19 pneumonitis. Doppler ultrasound screening of all four extremities was negative for DVT. He was started on IV UFH 8 units/kg/hr for DVT prophylaxis. One day after admission, aPTT was 32.9 seconds and within normal limits, but TEG demonstrated borderline hypercoagulability (Figure 3). The UFH dose was increased to 10 units/kg/hr IV. The same day, persistent heparin resistance prompted further dose escalation to 12 units/kg/hr. For the rest of his hospitalization, TEG guided goal-directed anticoagulation despite his hypercoagulable baseline (Figure 3), enabling us to achieve target aPTT of 40–50 seconds and *R* of 12–17 minutes with IV UFH 12–13 units/kg/hr. No thrombotic or hemorrhagic complications occurred prior to discharge.

## 5. Discussion

COVID-19 induces distinctive changes at the endothelium causing an endotheliitis [14]. The acute inflammatory response at the endothelium is marked by the permeating phagocytic cells releasing cytokines such as tumor necrosis factor-*α*, interleukin-1 (IL-1), IL-6, and IL-8 [15, 16]. This may be described as the “cytokine storm” in extreme cases. In turn, these cytokines activate factor XII, beginning the intrinsic coagulation cascade resulting in thrombin formation. Moreover, upregulation of plasminogen activator inhibitor 1 expression impairs fibrinolysis [17]. These pathophysiologic immune changes procure the hypercoagulable state often found in critically ill COVID-19 patients. However, as the cytokine storm recedes, these patients may develop a sensitivity to anticoagulation, which causes them to bleed. Hence, the risk of thrombosis in these patients evolves to a risk of bleed [18, 19].

The immunothrombotic changes induced by COVID-19 may not be unique. The endothelial surface may undergo similar pathology as found in ECMO patients as well as those with trauma-induced coagulopathy [10, 20]. The level of anticoagulation necessary to prevent thrombosis is likely a function of the magnitude of inflammation and the cytokine storm. As the cytokine storm wanes, intense monitoring and adjustment of the patient's hemostatic integrity with daily TEG and aPTT enable those responsible for COVID-19 anticoagulation to prevent thrombohemorrhagic complications in these critically ill patients. Based on the treatment of patients on ECMO, we elected to handle COVID-19 hypercoagulopathy with persistent monitoring by a coagulation team that followed the clinical pattern, TEG, and aPTT to guide anticoagulation [10]. We have found this strategy to be effective in guiding goal-directed anticoagulation of our COVID-19 patients, including the three examples above.

Limitations of TEG include the inability to replicate the effects of endothelium on hemostasis, negligible effects of endogenous anticoagulants, and a very low shear within a closed system. Furthermore, there has been recent discussion regarding the reliability of TEG to accurately detect and quantify hyperfibrinolysis [21].

## Figures and Tables

**Table 1:** Patient demographics.

	Patient 1	Patient 2	Patient 3
*Demographics*			
Age (years)	24	66	49
Sex	Male	Male	Male
BMI (kg/m^2^)	33.3	32.8	33.0

*Comorbidities*			
Cardiovascular disease	No	No	No
Chronic renal failure	Yes	No	No
Hypertension	Yes	Yes	Yes
Immunosuppression	No	No	No
Type II diabetes mellitus	No	No	No
Expiration	No	No	No

## Data Availability

No data were used to support the findings of this study.

## References

[1] American college of physicians (2021). *COVID-19: an ACP physician’s Guide + Resources. Chapter 12: Treatment: Supportive + Intensive Care*.

[2] Almskog L. M., Wikman A., Svensson J. (2021). Rotational thromboelastometry results are associated with care level in COVID-19. *Journal of Thrombosis and Thrombolysis*.

[3] Boscolo A., Spiezia L., Correale C (2020). Different hypercoagulable profiles in patients with COVID-19 admitted to the internal medicine ward and the intensive care unit. *Thrombosis and Haemostasis*.

[4] Musoke N., Lo K. B., Albano J. (2020). Anticoagulation and bleeding risk in patients with COVID-19. *Thrombosis Research*.

[5] Desborough M. J. R., Doyle A. J., Griffiths A., Retter A., Breen K. A., Hunt B. J. (2020). Image-proven thromboembolism in patients with severe COVID-19 in a tertiary critical care unit in the United Kingdom. *Thrombosis Research*.

[6] Al-Samkari H., Karp Leaf R. S., Dzik W. H. (2020). COVID-19 and coagulation: bleeding and thrombotic manifestations of SARS-CoV-2 infection. *Blood*.

[7] Fraissé M., Logre E., Pajot O., Mentec H., Plantefève G., Contou D. (2020). Thrombotic and hemorrhagic events in critically ill COVID-19 patients: a French monocenter retrospective study. *Critical Care*.

[8] NIH ACTIV Trial of Blood thinners pauses enrollment of critically ill COVID-19 patients. U.S. Department of Health and Human Services. https://www.nih.gov/news-events/news-releases/nih-activ-trial-blood-thinners-pauses-enrollment-critically-ill-covid-19-patients

[9] Gonzalez E., Moore E. E., Moore H. B. (2017). Management of trauma-induced coagulopathy with thrombelastography. *Critical Care Clinics*.

[10] Colman E., Yin E. B., Laine G. (2019). Evaluation of a heparin monitoring protocol for extracorporeal membrane oxygenation and review of the literature. *Journal of Thoracic Disease*.

[11] Panigada M., Bottino N., Tagliabue P. (2020). Hypercoagulability of COVID‐19 patients in intensive care unit: a report of thromboelastography findings and other parameters of hemostasis. *Journal of Thrombosis and Haemostasis*.

[12] Collett L. W., Gluck S., Strickland R. M., Reddi B. J. (2021). Evaluation of coagulation status using viscoelastic testing in intensive care patients with coronavirus disease 2019 (COVID-19): an observational point prevalence cohort study. *Australian Critical Care*.

[13] Yuriditsky E., Horowitz J. M., Merchan C. (2020). Thromboelastography profiles of critically ill patients with coronavirus disease 2019. *Critical Care Medicine*.

[14] Varge Z., Flammer A. J., Steiger P. (2020). Endothelial cell infection and endotheliitis in COVID-19. *Lancet*.

[15] Soy M., Keser G., Atagündüz P., Tabak F., Atagündüz I., Kayhan S. (2020). Cytokine storm in COVID-19: pathogenesis and overview of anti-inflammatory agents used in treatment. *Clinical Rheumatology*.

[16] Ye Q., Wang B., Mao J. (2020). The pathogenesis and treatment of the Cytokine Storm’ in COVID-19. *Journal of Infection*.

[17] Bhandary Y. P., Shetty S. K., Marudamuthu A. S. (2013). Regulation of lung injury and fibrosis by p53-mediated changes in urokinase and plasminogen activator inhibitor-1. *The American Journal of Pathology*.

[18] Han H., Yang L., Liu R. (2020). Prominent changes in blood coagulation of patients with SARS-CoV-2 infection. *Clinical Chemistry and Laboratory Medicine (CCLM)*.

[19] Bunch C. M., Thomas A. V., Stillson J. E. (2021). Preventing thrombohemorrhagic complications of heparinized COVID-19 patients using adjunctive thromboelastography: a retrospective study. *Journal of Clinical Medicine*.

[20] Moore H. B., Moore E. E., Neal M. D. (2019). Fibrinolysis shutdown in trauma. *Anesthesia & Analgesia*.

[21] Bareille M., Hardy M., Douxfils J. (2021). Viscoelastometric testing to assess hemostasis of COVID-19: a systematic review. *Journal of Clinical Medicine*.

